# Alcohol Modulates the Biogenesis and Composition of Microglia-Derived Exosomes

**DOI:** 10.3390/biology8020025

**Published:** 2019-04-27

**Authors:** Brennetta J. Crenshaw, Sanjay Kumar, Courtnee’ R. Bell, Leandra B. Jones, Sparkle D. Williams, Sabita N. Saldanha, Sameer Joshi, Rajnish Sahu, Brian Sims, Qiana L. Matthews

**Affiliations:** 1 Microbiology Program, Department of Biological Sciences, College of Science, Technology, Engineering and Mathematics, Alabama State University, Montgomery, AL 36104, USA; brcrenshaw@alasu.edu (B.J.C.); courtneerbell@yahoo.com (C.R.B.); ljones@alasu.edu (L.B.J.); 2 Departments of Pediatrics and Cell, Developmental and Integrative Biology, Division of Neonatology, University of Alabama at Birmingham, Birmingham, AL 35294, USA; skumar@peds.uab.edu (S.K.); swilliams@vt.edu (S.D.W.); bsims@peds.uab.edu (B.S.); 3 Department of Biological Sciences, College of Science, Technology, Engineering and Mathematics, Alabama State University, Montgomery, AL 36104, USA; ssaldanha@alasu.edu; 4 Center for Nanobiotechnology Research (CNBR), Alabama State University, Montgomery, AL 36104, USA; sjoshi@alasu.edu (S.J.); rsahu@alasu.edu (R.S.)

**Keywords:** exosome, biogenesis, microglia, tetraspanins, alcohol

## Abstract

Exosomes are small extracellular vesicles that have emerged as an important tool for intercellular communication. In the central nervous system, exosomes can mediate glia and neuronal communication. Once released from the donor cell, exosomes can act as discrete vesicles and travel to distant and proximal recipient cells to alter cellular function. Microglia cells secrete exosomes due to stress stimuli of alcohol abuse. The goal of this study was to investigate the effects of alcohol exposure on the biogenesis and composition of exosomes derived from microglia cell line BV-2. The BV-2 cells were cultured in exosome-free media and were either mock treated (control) or treated with 50 mM or 100 mM of alcohol for 48 and 72 h. Our results demonstrated that alcohol significantly impacted BV-2 cell morphology, viability, and protein content. Most importantly, our studies revealed that exosome biogenesis and composition was affected by alcohol treatment.

## 1. Introduction

Exosomes are small extracellular vesicles that are released from the cell after the fusion of multivesicular bodies with the plasma membrane [1]. They are found in biological fluids (i.e., breast milk, semen, urine, saliva) [2,3,4,5] and secreted by most cell types (i.e., glial, neurons, astrocytes, oligodendrocytes) [6,7,8,9]. Exosomes are involved in cell-to-cell communication. These vesicles transport nucleic acids such as miRNA, RNA, DNA, and proteins [10].

Exosomes have been shown to play a vital role in microglia function [11,12,13]. Microglia are the primary immune cells within the central nervous system (CNS) that regulate inflammatory processes during infection or cellular damage [9,12,14]. Upon activation, microglia undergo complex morphological and functional transitions, which includes increased phagocytosis, motility, and cytokine secretion [9]. Microglia secrete exosomes due to stress stimulated by alcohol consumption. Alcohol consumption disrupts signaling pathways (i.e., innate and adaptive immune responses) that are necessary for CNS homeostasis [14]. As a small molecule, alcohol can easily cross membrane barriers and reach different parts of the body very quickly, which can lead to total body alteration.

Alcohol interacts with brain receptors, interfering with communication between nerve cells, and suppressing the excitatory nerve pathway [15]. It has been documented that the abuse of alcohol impairs glial and astrocytic function [16]. Of important note, prenatal alcohol exposure alters the development of several brain regions, leading to structural and functional modifications in areas such as the cerebellum, cortex, and hippocampus [17], as well as white matter [18,19]. Alcohol-associated brain neuropathology can lead to behavior changes and impairments in learning, attention, executive functioning, and emotional regulation, which can seriously impact daily activities [20,21,22].

Microglia are vitally important brain-resident cells that are needed to support and maintain proper neural function. However, alcohol’s effects on microglia have recently gained attention [23]. Therefore, in this study, we investigated the effects of alcohol exposure on the biogenesis and composition of exosomes derived from microglia cell line BV-2. The BV-2 cell line used for these experiments is a murine cell line, which is often used as a substitute for primary microglia. The BV-2 cell line was cultured in exosome-free medium and was either not treated (control) or treated with 50 mM or 100 mM of EtOH for 48 and 72 h. Microglia cell morphology, cell viability, exosome biogenesis and composition was substantially altered by ethanol administration.

## 2. Materials and Methods

### 2.1. Cell Culture

Microglia cell line (BV-2) was a generous gift from Harald Neumann (University of Bonn LIFE and Brain Center, Bonn, Germany) [24]. BV-2 cells were cultured in Roswell Park Memorial Institute (RPMI) medium containing L-glutamine supplemented with 10% Corning Regular Fetal Bovine Serum (FBS) and 1% penicillin/streptomycin. RPMI exosome-free media was prepared using exosome-depleted FBS from System Biosciences (Biotechnology company in Palo Alto, Palo Alto, CA, USA).

### 2.2. Alcohol Dosing

BV-2 cells were plated at 5 × 10^5^ cells/per dish. The following day, the dishes were dosed with RPMI exosome-free media only (control treatment), and RPMI exosome-free media containing 200-proof 50 and 100 mM EtOH. The dosing duration was 48 and 72 h. Experiments were performed with four to six independent replicates.

### 2.3. Microglia Morphological Evaluation

The morphology of microglia cells were either control treated or treated with 200-proof 50 and 100 mM EtOH at 48 or 72 h. Morphology was observed using an Invitrogen EVOS FL system (Thermo Fisher Scientific, Waltham, MA, USA). The images were captured at a 20× magnification.

### 2.4. Cell Viability by Trypan Blue Exclusion

The cells were assessed for viability after control treatment or dosing with 200-proof 50 mM and 100 mM EtOH for 48 and 72 h. All media was removed. The cells were gently scraped with a cell scraper with 1.5 mL of non-sterile Phosphate Buffered Saline (PBS) solution. The solution was collected and gently mixed in 2.0 mL Eppendorf tubes. The cells were then counted and viability was assessed using trypan blue dye in Cell Countess (Invitrogen, Carlsbad, CA, USA).

### 2.5. Assessment of Cell Viability via Flow Cytometry

To access the percentage of cell death, 5 × 10^5^ cells/plate dish were seeded in 100-mm plates in 6 mL of complete culture medium. BV-2-microglia cells were treated with 50 and 100 mM of EtOH for 72 h. After incubation, cells were washed three times and incubated with 100 µL of annexin V-fluorescein-5-isothiocyanate (FITC) buffer for staining with annexin V-FITC and propidium iodide (PI) according to the manufacturer’s instructions. Annexin V-FITC (5 µL of 600 µg/mL) and propidium iodide (PI: 10 µL of 50 µg/mL) were added to each tube and incubated for 15 minutes (min) at room temperature (RT). For flow cytometric analysis, cells were suspended in 500 µL of Dulbecco’s Phosphate Buffered Saline (DPBS) and observed under a BD FACS Canto II Flow Cytometer (BD Biosciences, San Jose, CA, USA). PI-positive, annexin V-FITC-positive, and double-positive were counted as apoptotic cells, while PI–annexin V-FITC-negative cells were counted as live cells.

### 2.6. Assessment of Cell Cycle Progression

To evaluate the different phases of the cell cycle, cell-cycle analysis was performed as reported previously [25] with some changes. To accomplish this, 5 × 10^5^ cells/plate were treated with 50 and 100 mM of EtOH for 72 h. After incubation, cells were washed twice and fixed with 70% EtOH at 4 °C for 2–4 h. Cells were washed twice with PBS and treated with RNase A (50 µL of 100 µg/mL) for 15 min at RT. Next, intracellular DNA was labeled with propidium iodide solution (50 µg/mL) for 15 min at RT, protected from light, and analyzed by fluorescence-assisted cell sorting analysis (FACS) using a BD FACSCanto II Flow Cytometer (BD Biosciences, San Jose, CA, USA).

### 2.7. Exosome Isolation and Purification

The exosomes were isolated from RPMI exosome-free cell culture media. In brief, exosomes were isolated as previously described [26]. After collection, the media was spun down at 1300 revolutions per minute (rpm) at 4 °C for 10 min, using a Sorvall RT 6000 refrigerated centrifuge. The media was collected, and the pellet was discarded. The media was spun again at 3900 rpm at 4 °C for 10 min using a Sorvall RT 6000 refrigerated centrifuge (ThermoFisher Scientific, Waltham, MA, USA), and then filtered through a 10-mL syringe with a 25-mm syringe filter, with a porosity of 0.22 µm. PBS was added to the media and centrifuged at 10,800 rpm for 45 min in a SW41T1 swinging bucket rotor at 4 °C using a Beckman Coulter Optim L-70K Ultracentrifuge (Beckman Coulter, Brea, CA, USA). To collect the exosomes, the media was collected and centrifuged for 32,000 rpm for 70 min in a SW41T1 swinging bucket rotor at 4 °C using a Beckman Coulter Optima L-70K Ultracentrifuge. The exosome proteins were quantitated using the Bradford–Lowry quantitation method [27].

### 2.8. NanoSight Analysis

The exosome size distribution and particle number/mL were quantified by nanoparticle tracking analysis (NTA) (Nanosight-LM10, Malvern Instruments, Inc., Malvern, UK). The samples were diluted in 1X PBS and placed inside of a 0.3-mL disposable syringe. The NTA analyzes the size of the particle based on Brownian motion of the particle as well as scattered light, and can analyze a 1000-times-diluted sample compared to dynamic light scattering (DLS). Mean values were recorded and analyzed for each given reading frame of the five independent experiments.

### 2.9. Dot Blot Analysis

Exosomes and lysates were evaluated via dot blot analysis; 5 µg of exosome protein or lysate was boiled and bound to nitrocellulose membranes for 10 min. Samples were blocked in a Pierce Fast-Blocker with 0.09% Tween-20 for 5 min. After blocking, primary antibodies (CD63 (1:500, Santa Cruz Biotechnology, Dallas, TX, USA); Rab 7 (1:500, ThermoFisher Scientific, Waltham, MA, USA) or cleaved caspse-3 (1:500, R&D system, Minneapolis, MN, USA) were added to the samples for incubation. Samples were incubated for 1 h at RT. Nitrocellulose blots were washed three times with 0.09% Tween-20 in 1× PBS for 10 min. Goat anti-rabbit heavy and light chain (H+L) secondary antibody Horseradish peroxidase (HRP) (1:1000 Novus Biologicals, Centennial, CO, USA) was added in blocking solution (0.09% Tween-20 in 1× PBS) for 1 h, shaking at RT. The blots were washed three times with 0.09% Tween-20 in 1× PBS for 10 min. The nitrocellulose membranes were developed using SuperSignal West Femto Maximum Sensitivity Substrate (Thermo Scientific, Waltham, MA, USA). The signals were developed on a Bio-Rad ChemiDoc XRS+ System (BioRad Laboratories, Hercules, CA, USA).

### 2.10. Enzyme-Linked Immunosorbent Assay (ELISA)

Enzyme-Linked Immunosorbent Assay (ELISA) was performed to determine the proteins found in or on the exosomes prior to and after EtOH exposure. Exosomes that were previously collected in pellet form were mixed via hand pipetting, and 30 µg of these exosomes were bound in 100 µL of bicarbonate buffer in a 96-well plate and placed at 4 °C overnight. The exosomes were washed in 200 µL of washing buffer (0.05% Tween-20 in PBS) 4 times. The plate was then blocked with 100 µL of blocking solution (5% non-fat dry milk and 0.05% Tween-20 in 1× PBS) for 1 h. Primary antibodies were added to each assigned well accordingly and placed at 4 °C for 2 h. The plates were again washed with washing buffer 4 times and blocked with 100 µL of blocking buffer at RT for 30 min. The secondary antibodies were added to each assigned well accordingly and placed at 4 °C for 2 h followed by washing with buffer (0.05% Tween-20 in PBS); 100 µL of ELISA substrate buffer containing 1 mg/mL *o*-Phenylenediamine dihydrochloride (OPD) (Sigma-Aldrich, St. Louis, MO, USA) was added to the 96-well plate and incubated for 30 min. The optical density (OD) of the colored solution was recorded using the GeneMate UNIREAD 800 plate reader (BioExpress, Kaysville, UT, USA).

### 2.11. Statistical Significance

Descriptive statistics were calculated to study various variables of importance (i.e., means). Statistics were performed using one-way ANOVA with post hoc Tukey’s analysis. Statistical significance was defined as follows: (*) *p* ≤ 0.05; (**) *p* ≤ 0.01; (***) *p* ≤ 0.001 or (****) *p* ≤ 0.0001.

## 3. Results

### 3.1. Percent Viability of BV-2 Cells after Alcohol Administration

**Figure 1:**
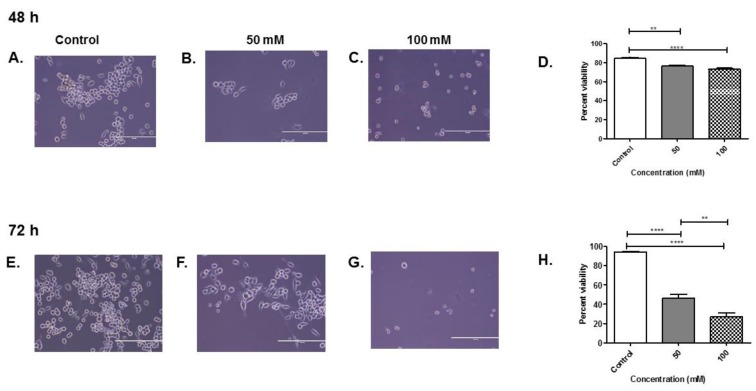
Effects of EtOH on microglial cell line BV-2 cells. BV-2 cells were treated with ETOH for (**A**–**C**) 48 or (**E**–**G**) 72 h. (**D**,**H**) At 48 and 72 h, EtOH administration of cell viability was assessed by trypan blue exclusion assay. Photos were captured at 20× magnification (**A–C**,**E–G**). The scale bar = 400 µm. Data are presented as mean ± SEM. Significant differences were determined using one-way ANOVA with post hoc Tukey’s analysis. Significance is defined as (**) *p* ≤ 0.01, (****) *p* ≤ 0.0001.

To determine the effect of alcohol on the microglia cell line BV-2, the following experiments were performed. Microglia BV-2 cells were either untreated (control) or treated with EtOH at 50 and 100 mM in exosome-free medium for 48 and 72 h. The cell morphology was examined through the digital inverted microscopy (Figure 1A–C,E–G). At both 48 and 72 h, 50 and 100 mM EtOH exposure, cells were less dense as compared with the control cells (no treatment) due to a lack of proliferation (Figure 1A–C,E–G). At both 48 and 72 h, 100 mM EtOH exposure, cells shrunk compared with the control cells (no treatment) (Figure 1A,C,E,G).

Cell viability was evaluated using the trypan blue exclusion assay. The trypan blue exclusion assay was performed to determine the number of viable cells present in the cell suspension after EtOH administration at 48 and 72 h (Figure 1D,H). At 48 h treatment with 50 and 100 mM EtOH, cell viability was decreased significantly to 74% and 73% in both treatment groups (*p* ≤ 0.01, *p* ≤ 0.0001) (Figure 1D). Furthermore, at 72 h treatment, with 50 and 100 mM EtOH, cell viability was significantly decreased to ~50% viability (*p* ≤ 0.0001) and ~25% viability (*p* ≤ 0.0001), compared with the control treatment, as seen in Figure 1H. Our results indicate that alcohol exposure at 48 and 72 h reduced the viability of BV-2 cells.

### 3.2. Apoptotic Status of BV-2 Cells Treated with EtOH

**Figure 2:**
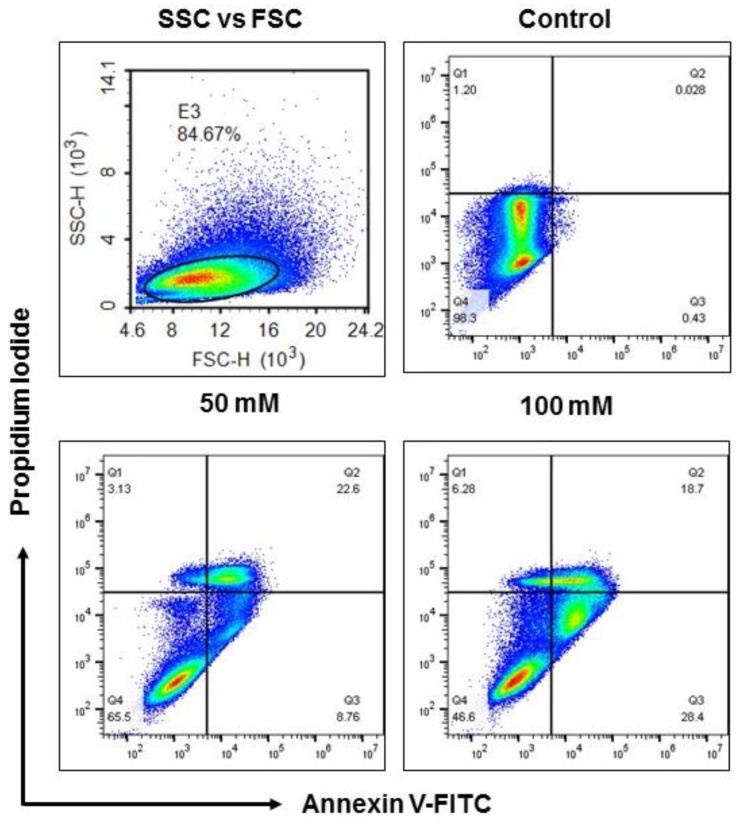
EtOH treatment reduced BV-2 cell viability in a dose-dependent manner. BV-2 cells were treated with 50 and 100 mM of EtOH at 72 h and cell viability was tested. Cells were washed and labeled with Annexin V-FITC and PI to discriminate apoptotic and healthy cells. For this experiment, live cells (E3: 84.67%) were gated to determine the percentage of early (Q3), late (Q2) and apoptotic (Q1) cells after EtOH exposure. Early apoptotic (Q3) and apoptotic (Q1) cell were higher when dosed with 100 mM; however, 50 mM showed higher a percentage of late apoptotic (Q2) cells. FSC: Forward Scatter; SSC: Side Scatter.

To determine if the cells were undergoing apoptosis, Annexin V-FITC and PI staining was performed. This staining revealed externalization of phosphatidylserine (PS) and chromatin condensation, one of the hallmarks of apoptosis or programmed cell death (Figure 2). Incubation of BV-2 cells with 100 mM of EtOH demonstrated a considerable number of BV-2 cells in early apoptotic phase (these cells exposed PS to the outer leaflet that has great affinity to annexin V, and can be detected in the FITC channel using FITC-conjugated annexin V) and apoptotic (these cells showed fragmentation of genomic DNA, which can be detected using a DNA labeling dye such as PI) phases as compared to the exposure of 50 mM EtOH and without any treatment. While 50 mM EtOH exposure showed a higher number of late apoptotic cells (PS externalization and DNA fragmentation), suggesting alcohol administration induced apoptosis of BV-2 microglia cells (Figure 2).

### 3.3. Alcohol Exposure Modulates Cell Cycle Progression in BV-2 Microglia Cells

**Figure 3:**
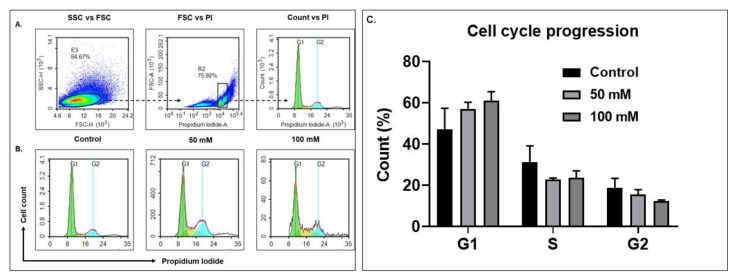
EtOH exposure modulate cell-cycle progression in BV-2 cell lines. For cell-cycle analysis at 72-h EtOH treatment, intracellular DNA was labeled with PI possessing a high affinity with DNA, and was used to detect DNA content during cell division. (**A**) Schematic representation (live cells were gated to exclude duplets cells (R2: 75.92%) and these cells were used to plot a cell-cycle histogram (count vs. PI)). (**B**) Cell-cycle phases (G1, S and G2) post-exposure with 50 and 100 mM, and (**C**). Comparative histogram of the different phases of the cell cycle (Control, 50 and 100 mM).

To further confirm the apoptotic characteristic of BV-2 cells after exposure with alcohol, cell-cycle analysis was carried out. BV-2 cells stained with PI demonstrated a percentage of cells in G1, S and G2 phases, characterized as fragmented DNA, which was determined by FACS. At 100 mM EtOH there was an accumulation of G1-phase or G1-arrest cells, the latter of which is an indicator of apoptosis. The proportion of G1-arrest cases increased as compared with the cells without treatment at 72 h (Figure 3). However, synthesis/S-phase and G2-phase cells did not show a remarkable change after the exposure with 50 and 100 mM of EtOH treatment (Figure 3), suggesting alcohol treatment sensitized BV-2 cells in the G1 phase and induced apoptosis.

### 3.4. Size Distribution and Quantity of BV-2-Derived Exosomes after Alcohol Administration

**Figure 4:**
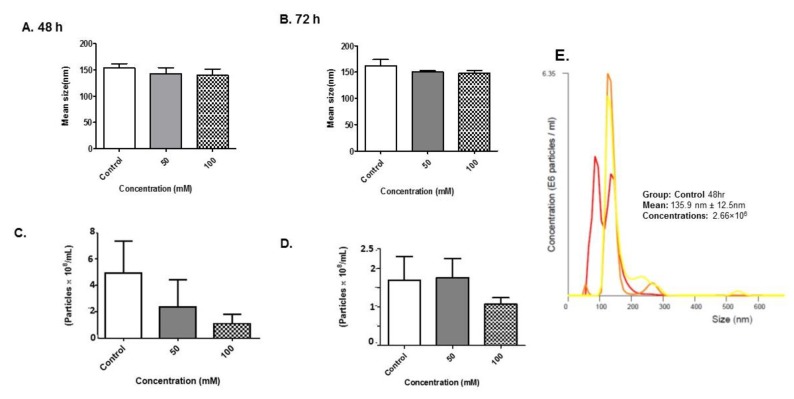
Nanoparticle tracking analyses validation of BV-2-derived exosomes. NTA-generated (**A**,**B**) size and (**C**,**D**) concentration of vesicles obtained from five independent experiments after EtOH administration. (**E**) Histogram plot of control exosomes collected at 48 h. The different colored lines represent different pools of exosomes.

To determine the effect of alcohol on BV-2-derived exosomes, BV-2 cells were cultured in exosome-free media and treated with alcohol (Control, 50 and 100 mM) for 48 and 72 h. The exosomes released into the cultured media were isolated and purified for biophysical analyses. Quantitation of exosome size was determined using NTA (Figure 4A–E). The exosomes were visualized by light scattering using a laser scattering microscope with a video camera. A video was taken, and the NTA software tracked the Brownian motion of the individual vesicles, calculating their size and concentration. Analysis of exosomes by NTA revealed exosomes that were approximately the same size in diameter at the 48- and 72-h time points (Figure 4A–B,E). Exosome sizes averaged under ~150 nm throughout all experiments.

Analysis of exosomes by NTA revealed a slight decrease in the total number of exosomes produced between the control group and treatment groups at 48 h administration (Figure 4C). Representative graphs of control exosomes at 48 and 72 h EtOH administration indicated that BV-2-derived exosomes had a concentration of 4.08 × 10^8^ particles/mL and 1.50 × 10^8^ particles/mL (Figure 4C–D). In summary, EtOH administration effects exosome particle numbers markedly at early time points.

### 3.5. Expression of Rab Proteins

**Figure 5:**
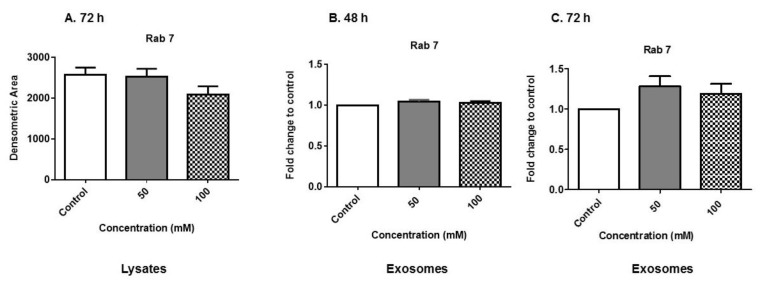
Expression of exosome biogenesis and secretory proteins. (**A**) Cell lysates (at 72 h EtOH administration) and (**B**,**C**) exosome proteins (at 48 and 72 h EtOH administration) were evaluated for Rab 7 expression. In order to obtain quantitative results, cell lysates were subjected to dot blot analysis and exosomes were subjected to ELISA. Experiments involving dot blot analysis and ELISA were performed with four to six independent replicates.

Rabs are a group of GTPase proteins that are involved in membrane trafficking, vesicle formation and secretion. We evaluated Rab 7 expression in cell lysates and exosomes after EtOH administration. Lysates herein the manuscript refers to cell extract and not exosomal extract. Lysates at the 72-h time point were selected for evaluation to ensure protein expression. Using dot blot analysis, we observed a slight decrease in Rab 7 protein expression in cell lysates at 72 h/100 mM EtOH treatment (Figure 5A). We observed Rab 7 protein expression in exosomes at 48 h, and this expression remained constant in exosomes that were derived after EtOH administration at either EtOH concentration (Figure 5B). However, at the 72-h time point, Rab 7 protein expression inversely increased in exosomes (Figure 5C) as compared with cell lysates (Figure 5A). Rab proteins were likely reduced in the cell lysate after EtOH treatment because they are packaged in the exosomes—it is well documented that Rab 7 is found in some types of exosomes [28,29]. This data suggests the impact of alcohol on exosome biogenesis.

### 3.6. Analyses of Exosome-Associated Proteins

**Figure 6:**
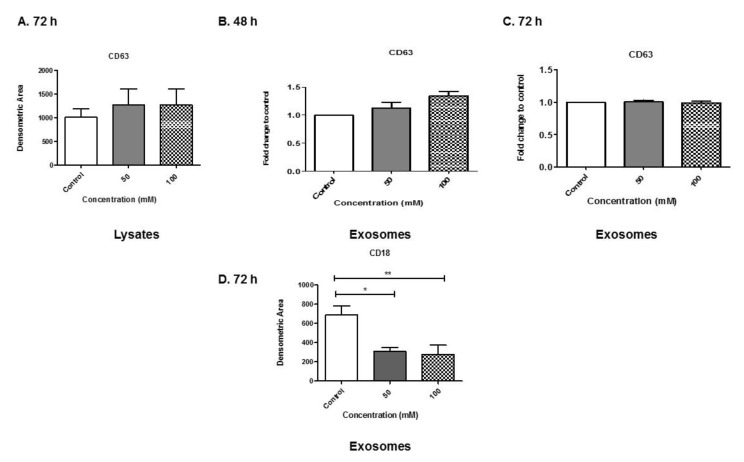
Expression of exosomal marker proteins. (**A**) Cell lysates (at 72 h EtOH administration) and (**B**–**D**) exosome proteins (at 48 and 72 h EtOH administration) were evaluated for cluster of differentiation (CD)63 or CD18 expression. In order to obtain quantitative results, cell lysates were subjected to dot blot analysis and exosomes were subjected to ELISA. Experiments involving dot blot analysis and ELISA were performed with four to six independent replicates. Data are presented as mean ± SEM. Significant differences were determined using one-way ANOVA with post hoc Tukey’s analysis. Significance is defined as (*) *p* ≤ 0.05, (**) *p* ≤ 0.01.

To analyze proteins associated with exosomes, BV-2-derived exosomes were evaluated. We first evaluated the levels of tetraspanin-associated exosomal proteins cluster of differentiation (CD)63 in lysates and exosomes (Figure 6A–C). CD63 is a classic exosome marker. All of the BV-2 lysates expressed CD63 (Figure 6A). At 48 h, there was an increasing trend of CD63 on exosomes at 100 mM EtOH administration compared with control (Figure 6B). At 72 h, CD63 levels were constant within all exosome samples (Figure 6C). In addition to CD63, CD18 was evaluated. CD18 was evaluated because it is a marker for microglial cells as well as some other immune cell types. Microglia express many other surface markers such as CD18, CD11a, CD11c, etc. [30]. At 72 h EtOH exposure, CD18 levels were significantly decreased in BV-2 derived exosomes (Figure 6D). Importantly, this data illustrates that EtOH administration modulates exosome markers. We speculate that this result can regulate exosome trafficking and cell-to-cell communication.

### 3.7. Alcohol Dosing Increases Heat Shock Proteins within Exosomes

**Figure 7:**
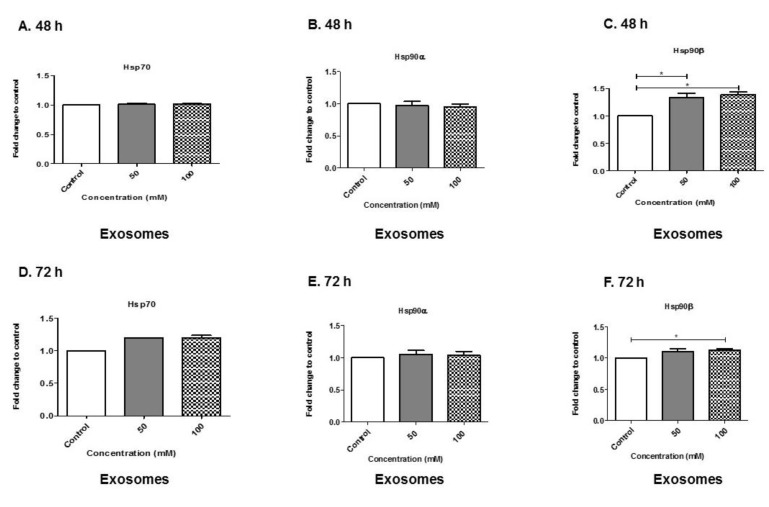
Production of heat shock proteins within BV-2-derived exosomes. (**A**,**D**) Hsp70, (**B**,**E**) Hsp90α and (**C**,**F**) Hsp90β were expressed in exosomes after EtOH dosing at 48 or 72 h using ELISA. Experiments involving ELISA were performed with four to six independent replicates. Data are presented as mean ± SEM. Significant differences were determined using one-way ANOVA with post hoc Tukey’s analysis. Significance is defined as (*) *p* ≤ 0.05.

Heat shock proteins (Hsp) are molecular chaperone proteins that facilitate the synthesis and folding of proteins. They are induced in response to environmental stimuli and stressors [31,32]. Therefore, we evaluated the levels of BV-2-derived exosomes expressing heat shock proteins 70 and 90, alpha (α) and beta (β) (Figure 7A–F). BV-2-derived exosomes expressing Hsp70 showed no net change at 48 h EtOH exposure (Figure 7A). However, at the 72-h time point, BV-2-derived exosomes expressing Hsp70 showed a slight increase at 50 and 100 mM EtOH administration compared with the control (Figure 7D).

Hsp90α has been found in exosomes derived from immune cells and other cell types [28,33,34,35,36]. BV-2-derived exosomes expressing Hsp90α showed no net change at 48 or 72 h (Figure 7B,E). However, BV-2-derived exosomes showed a significant increase in Hsp90β at 50 mM EtOH administration (*p* ≤ 0.05) compared with control treatment at the 48-h time point (Figure 7C). In addition, BV-2-derived exosomes expressing Hsp90β showed a significant increase at 100 mM EtOH (*p* ≤ 0.05) administration compared to no treatment (Figure 7C) at 48 h. At 72 h, BV-2-derived exosomes expressing Hsp90β were significantly increased at 100 mM EtOH administration (*p* ≤ 0.05), as compared with control treatment (Figure 7F). Our results indicate that alcohol increased the production of heat shock proteins and their subsequent packing into exosomes.

### 3.8. Alcohol Dosing Alters Apoptotic Proteins

**Figure 8:**
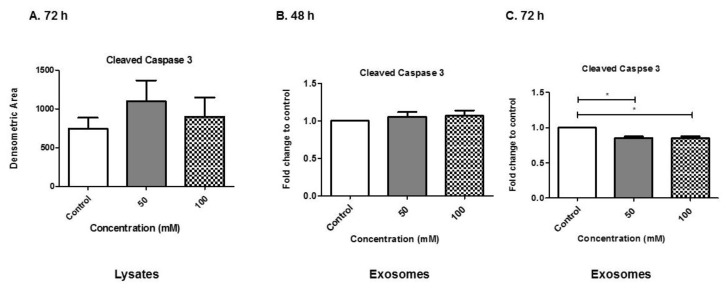
Cleaved caspase 3 in BV-2-derived exosomes. Cleaved caspase 3 was expressed in lysates and exosomes after EtOH dosing and detected using dot blot and ELISA. (**A**) Cell lysates (at 72 h EtOH administration) and (**B**,**C**) exosome proteins (at 48 and 72 h EtOH administration) were evaluated for cleaved caspase 3 expression. Experiments involving ELISA were performed with four to six independent replicates. Significant differences were determined using one-way ANOVA with post hoc Tukey’s analysis. Significance is defined as (*) *p* ≤ 0.05.

Caspases are involved in regulating cell death in cells undergoing stress. Caspases are activated in a variety of conditions (i.e., infections and chemical stimuli). Levels of cleaved caspase 3 in BV-2 cells and BV-2-derived exosomes post-EtOH were evaluated. The presence of cleaved caspase 3 indicates the active form of the protein, which can then lead to an apoptotic cascade. Cell lysates from BV-2 cells was evaluated after 72-h EtOH administration (Figure 8A). There was an increase in cleaved caspase 3 at the 72-h time point. At 48 h EtOH administration we evaluated exosomes carrying cleaved caspase 3 and there was no notable difference of cleaved caspase 3 detected (Figure 8B). However, at the 72-h time point, we observed that the administration of EtOH (50 or 100 mM) significantly decreased the amounts of cleaved caspase 3 packaged within exosomes (*p* ≤ 0.05, *p* ≤ 0.05) (Figure 8C). Our results indicate that alcohol modulates trafficking of caspase 3 within exosomes. This result is similar to the reciprocal relationship observed in Rab 7 protein expression seen in lysates and exosomes (Figure 5).

## 4. Discussion

Currently, alcohol is one of the most abused drugs in the world. Alcohol has a tremendous impact on organs in the body and overall health. The impact of alcohol on the brain is tremendous, ranging from addiction, dependency and loss of critical motor skills and function [37]. Specifically, EtOH consumption during pregnancy has been associated with irreversible phenotypic abnormalities, generally referred to as fetal alcohol spectrum disorders. Although there has been progress in this area, there are significant questions that still remain. Regarding these major adverse outcomes, there are multiple issues that are occurring at the cellular and subcellular levels. Mao et al. demonstrated that EtOH prevents trafficking of Sonic hedgehog protein into transport vesicles from the Golgi to the plasma membrane [38]. This finding may implicate EtOH in modulating other mechanisms involving micro- and nanostructures. One such nanostructure of importance is exosomes, due to their role in cell-to-cell communication.

Exosomes have been confirmed to be released from many cell types, including neurons, astrocytes and microglia [7,39]. Microglia are the resident brain macrophage. Microglia maintain tissue homeostasis, provide the first line of defense against infection and brain injury and promote tissue repair [39]. We have begun to know the impact of alcohol consumption related to exosome biogenesis in cells derived from the liver and heart. However, little is known related to alcohol consumption and exosome biogenesis in the brain. In this study, we investigated the effects of alcohol exposure on the biogenesis and composition of exosomes derived from microglia cell line BV-2. The effects of alcohol on microglia are poorly understood.

Our results show that alcohol administration significantly sensitized BV-2 cells; the results were confirmed via morphological analysis and a noticeable reduction in BV-2 viability at 50 and 100 mM concentrations at 48 and 72 h exposure when compared to control (Figure 1). Concentrations of 50 and 100 mM EtOH correspond to low levels of alcohol consumption and legal intoxication (in most states), or the consumption of a high number of alcoholic drinks [40]. In addition, BV-2 viability after 72 h was dramatically decreased between 50 and 100 mM concentrations (Figure 1). Exosomes were isolated using our standard methods and characterized after BV-2 cells were dosed with EtOH [26,41]. The data illustrate that cells were undergoing apoptosis and cell-cycle arrest (Figure 2 and Figure 3). NTA revealed a slight decrease in the particle numbers observed at 48 h after dosing (Figure 4C). This finding needs to be further investigated to determine how particle numbers modulate cell protection or programmed cell death. Our studies indicated that exosomes remain intact in the presence of EtOH after administration [42]. 

At 72 h alcohol administration in cell lysates, we observed a decrease in Rab 7 protein expression (Figure 5A). This finding inversely correlates with an increase in Rab 7 expression and packaging in exosomes at 72 h (Figure 5C). Rab proteins are likely reduced in cell lysate after EtOH treatment because they are trafficked into the exosomes. It is well documented that Rab 7 is found in some types of exosomes. This finding indicates that exosome biogenesis is impacted by EtOH administration in BV-2 cells (Figure 5). These findings are novel in our studies because Rabs have been shown to play an important role in vesicle trafficking [43]. Rab 7 expression particles have two possible fates, with two physiological consequences [29,43]. The first fate is that multivesicular bodies fuse with lysosomes (generating the endolysosomal compartment), leading to the degradation of its vesicular contents. The second fate is that multivesicular bodies traffic to and fuse with plasma membranes, leading to the release of their intraluminal vesicles as exosomes [29]. These exosomes carry cell-specific active macromolecules; therefore, what determines either fate is not completely known [44]. Notably, Rab 7 has been found in other immune-derived exosomes, such as dendritic cell-derived exosomes [10]. In separate experiments, we also observed the presence of Rab 11, 27A and 35 in BV-2 lysates [42].

When evaluating BV-2-derived exosomes for exosome-associated markers, we observed that BV-2-derived exosomes presented CD63 via dot blot and ELISA (Figure 6). CD63 is a classic exosome marker. We observed a slight increase in CD63 on BV-2-derived exosomes after BV-2 cells were treated with 100 mM EtOH at 48 h. This finding suggests that CD63 is present in BV-2-derived exosomes, which is consistent with other reports that these tetraspanins have broad tissue distribution [45]. In addition, we evaluated CD18 expression in exosomes, and observed significant decreases in CD18 as a result of EtOH treatment, which is of interest and requires further investigation. CD18 is found in a variety of cells including those with a lineage of immunity. CD18 pairs with CD11, and can lead to activation of the cell [30]. We speculate that CD18 expression remains constant or increases in the cell lysate similar to what is observed with cleaved caspase 3 (Figure 8A).

The importance of tetraspanins is that they can form tetraspanin-enriched microdomains (TEMs), which are multifunctional. TEMs (a) function as mediators of extracellular vesicle biogenesis, (b) aid with the selection of exosome cargo (DNA, proteins, miRNAs), (c) function in the binding and uptake of exosomes by target cells and (d) allow exosomes to present antigens in the context of an immune response [45]. In the context of EtOH treatment, our study only evaluated CD63 as a classic tetraspanin. However, more investigation is needed in regards to EtOH and its ability to modulate other tetraspanins (i.e., CD9, CD81, CD82, etc.) [46] and the TEMs on BV-2-derived exosomes.

Hsp is an indispensable family of protective proteins [47]. Hsps target proteins for degradation by means of ubiquitination. Hsps also protect cells against injury, such as heat stress or hypoxia-reoxygenation [47], as well as protecting against some substances such as geranylgeranylacetone [48], glutamine [49] and others. Based on these facts, we investigated EtOH administration and its ability to modulate Hsp within exosomes. Specifically, we evaluated exosomes for Hsp60, Hsp70, Hsp90α and Hsp90β. In our studies, we did not detect Hsp60 in BV-2-derived exosomes with the assays used [42]. This finding is of importance to the knowledge of EtOH-derived exosomes. Malik et al. studied cardiac myocyte exosomes, specifically EtOH-derived exosomes, and found that ethanol did not affect the stability of cardiac myocyte-derived exosomes, but that it did greatly increase the production of these exosomes. Furthermore, their study found that Hsp60 is predominantly linked with the exosomal membranes [47,50].

Stressful events in astrocytes, such as heat or oxidative stress, were shown to release exosomes carrying Hsp70 and synapsin 1, which have a pro-survival effect on neurons [51]. Furthermore, Hsp70 protects brain cells against ischemia and other stressors [52]. One mechanism of Hsp70 protection may be its ability to prevent damaging pro-inflammatory responses [53]. Our data indicate that Hsp70 was in BV-2-derived exosomes at both time points (Figure 7A,D). Past studies have indicated that Hsp70 can act as a ligand for the Toll-like receptors existing on immune cells, including microglia [54]. For Hsp to bind Toll-like receptors, it has to be extracellular [55]. Experiments from other groups have shown that, in the presence of controlled cell lysis, Hsp secretion occurs in the absence or independence of cell death, suggesting that the release of Hsp occurs by active mechanisms. Exosomes are one of the accepted mechanisms of exosome secretion [56].

Hsp90 is another known molecular chaperone, and it regulates Hsp70 induction [55]. The impact of inhibition of Hsp90 on the brain is still unclear. Interestingly, we observed in our study that BV-2-derived exosomes collected at 48 h of EtOH exposure yielded exosomes that contained significant amounts of Hsp90β (*p* ≤ 0.05, *p* ≤ 0.05) (Figure 7C). Similarly, we observed that BV-2-derived exosomes collected at 72 h of EtOH exposure yielded exosomes containing significant amounts of Hsp90β (*p* ≤ 0.05) (Figure 7F). Hsp90 is one of the most abundant proteins in eukaryotes, comprising as much as 1%–2% of the total cellular protein. Hsp90 increases about two-fold following environmental stress [57,58,59,60]. Besides its vital role in maintaining normal tissue homeostasis, Hsp90 is an ATP-dependent molecular chaperone that plays a role in stabilizing and activating more than 200 client proteins, many of which are essential for constitutive cell signaling and adaptive responses to stressors [61,62,63]. In human cells, Hsp90 can be found in the cytosol, nucleoplasm, endoplasmic reticulum and mitochondria [64]. In higher eukaryotes, three Hsp90 species are expressed in the cytosol, Hsp90α, Hsp90β and (TRAP [65] or Hsp75 [66]). The observation that Hsp90β is found in great abundance in BV-2-derived exosomes after EtOH administration is of significance. The abundance of Hsp90β could be modulating apoptotic cascade directly or indirectly, as observed in Figure 1, Figure 2 and Figure 8.

## 5. Conclusions

Overall these findings, have several implications for neuroprotection in the brain after alcohol consumption. EtOH impacts cells of the CNS and the brain. Exosome modulation is likely to have an impact on these phenomena. More investigation is needed in this area.
